# Utility of routine laboratory preoperative tests based on previous results: Time to give up

**DOI:** 10.11613/BM.2017.030902

**Published:** 2017-08-28

**Authors:** Enrique Rodríguez–Borja, Africa Corchon–Peyrallo, Gerardo Aguilar–Aguilar, Arturo Carratala–Calvo

**Affiliations:** 1 Laboratory of Biochemistry, Hospital Clínico Universitario Valencia, Valencia, Spain; 2 Service of Anesthesia, Hospital Clínico Universitario Valencia, Valencia, Spain

**Keywords:** cost-effectiveness, preoperative care, patient safety, routine diagnostic tests

## Abstract

**Introduction:**

The usefulness and cost-effectiveness of routine laboratory preoperative tests (POTs) have been challenged recently. In fact, the American Society of Anesthesiologists (ASA) Task Force has stated that test results obtained from the medical record within 6 months of surgery generally are mostly acceptable. The aim of our study was to evaluate the degree of utility of POTs and their clinical benefit based on this recommendation.

**Material and methods:**

We studied retrospectively every routine POT request from 8 randomly selected weeks in 2016. Every POT contained glucose, creatinine, haemoglobin and coagulation tests (PT-INR). Each pathological result for these tests was registered and classified as “expected” (if previous pathological result within 6 months existed for that test) or “unexpected” (if previous pathological result within 6 months did not exist for that test). Results of ASA physical status classification (a system for assessing the fitness of patients before surgery) and changes in patient management after POTs were retrieved from medical history as well.

**Results:**

A total of 4516 tests (from 1129 patients) were analysed and 498 results were found pathological (11%). Of these, 403 were expected (8.9%) and 95 unexpected (2.1%). There was not any change in anaesthetic management for any patient due to these findings.

**Conclusions:**

Routine POTs are an inefficient and low-value service. POTs have to be always ordered selectively after a previous consideration of specific information obtained from several sources (medical records, interviews, examinations, type of surgery) and only if the information obtained will result in changes in the management of the patient.

## Introduction

According to the Practice Advisory for Preanesthesia Evaluation by the American Society of Anesthesiologists (ASA) Task Force published in 2012, pre-anaesthesia evaluation consists of the consideration of information from several sources that may include not only patient´s medical records, but interview, physical examination and findings from medical tests and evaluations (*1*). Theoretically, preoperative tests (POTs) are aimed at discovering a disorder and/or verifying an already known disease that may affect preoperative anaesthetic care. These assessments may be used to formulate specific plans or alternatives for preoperative anaesthetic care and therefore reduce morbidity associated with surgical-anaesthetic procedures (*1*).

The preoperative investigations include routine laboratory tests (metabolic panel, complete blood count, coagulation studies) done in the absence of any specific clinical indication or purpose. The appropriateness of this routine laboratory testing has been challenged in recent years. In fact, its usefulness and cost-effectiveness have been questioned, as the probability of finding a significant abnormality is small (*2*). Besides, routine POTs rarely change management, involve a sizable cost and may cause harm to patients (*3*). We assume that a normal test value is set arbitrarily based on 95% confidence interval; therefore up to 5% of normal individuals may have a value outside of the reference interval. Moreover, if we consider that tests are independent of each other, then the greater the number the tests ordered, the greater the chance of obtaining a result outside of the reference interval in a healthy patient (*4*). Screening tests in an asymptomatic population should only be done in patients with a significant and prevalent condition.

In 2013, ASA, through the Choosing Wisely campaign, gave the following recommendation applicable to all outpatient surgery: “Don´t obtain baseline laboratory studies in patients without significant systemic disease (ASA physical status I or II) undergoing low-risk surgery – specifically complete blood count, basic metabolic panel and coagulation studies when blood loss or fluid shifts is/are expected to be minimal”. By the same token, the ASA Task Force has stated that test results obtained from the medical record within 6 months of surgery generally are acceptable if the patient´s medical history has not changed substantially (*1*).

The purpose of our study was to evaluate the degree of appropriateness (based on previous test results within 6 months and patient physical status) and the clinical benefit (based on a change in patient management) of routine laboratory POTs performed during pre-anaesthetic evaluation in outpatients undergoing surgery at our hospital.

## Materials and methods

### Study design

The study was conducted in the Biochemistry Laboratory of Valencia´s Clinic Hospital. The centre serves around 350,000 people in Valencia´s metropolitan area. We studied retrospectively, through an enquiry to our Laboratory Information System (LIMS) (Gestlab. Cointec^©^ Spain), every POT request from a randomly selected period of eight weeks in 2016. POT activity and results were retrieved from every outpatient attending pre-anaesthesia evaluation during those weeks.

### Methods

Routine POT is a test profile ordered through a specific panel and contains glucose, creatinine, complete blood count (CBC) and prothrombin time with INR. Cost of our POT was estimated at 22 € according to our Regional Health Taxes Law.

Each pathological or outside of reference interval (ORI: out of the age- or sex-related laboratory reference values) for glucose, creatinine, haemoglobin and coagulation test (PT-INR) was registered. Additionally, every ORI test was classified as “expected” (if there was another previous pathological result within 6 months before POT) or “unexpected” (if there was not another previous pathological result within 6 months before POT) based on LIMS medical records. Haemoglobin was singled out from CBC because, in practice, is the main haematological parameter of relevance for our anaesthesiologists in their preoperative activity.

Results of ASA score and changes in patient management after POTs were retrieved from medical history database for every patient (children and adults). The ASA physical status (ASA-PS) classification system assesses the fitness of patients before surgery. The four categories used in our study were: ASA-PS I (Normal healthy patient), ASA-PS II (Patients with mild systemic disease), ASA-PS III (Patients with severe systemic disease) and ASA-PS IV (Patients with severe systemic disease that is a constant threat to life).

## Results

We performed POTs on 1129 patients (579 females, 550 males). Median age was 60 years (range 2 - 93). A total of 227, 609, 252 and 41 patients were classified as ASA-PS I, II, III and IV, respectively. Approximately 74% of the patients were classified as ASA-PS I or II. In total, 4516 tests were analysed and 498 results were ORI (11%). Of these pathological results, 403 were expected (8.9%) and 95 were unexpected (2.1%). Of these 403 expected results, 184 (4.1%) were from ASA I-II patients and 219 (4.8%) from ASA-PS III-IV patients, with high glucose concentrations being the most frequent expected pathological result. Furthermore, of the 95 unexpected results, 58 (1.3%) were from ASA-PS I-II patients and 37 (0.8%) from ASA-PS III-IV and low haemoglobin concentrations were the most frequent unexpected pathological test (Table 1).

**Table 1:** Comparison of outside reference interval (ORI) expected and unexpected test results

**ASA-PS score**	**Patients, N**	**PT-INR results, N**		**Haemoglobin results, N**		**Glucose results, N**		**Creatinine results, N**	
**Exp**	**Une**	**Exp**	**Une**	**Exp**	**Une**	**Exp**	**Une**
**I**	227	0	0	7	8	2	4	3	**1**
**II**	609	9	0	40	10	99	19	24	**16**
**III**	252	24	1	28	17	86	5	38	**11**
**IV**	41	5	0	9	2	14	1	15	**0**
**TOTAL**	**1129**	**38**	**1**	**84**	**37**	**201**	**29**	**80**	**28**
ASA – physical status according to the American Society of Anesthesiologists. Exp - expected test result. Une - unexpected test result.									

There was only one unexpected pathological test for PT-INR in a patient ASA-PS III. The patient came from another medical department and had no previous medical records in our hospital, but he was under treatment with acenocumarol at that time. No unexpected pathological creatinine result higher than 131 µmol/L and no unexpected pathological haemoglobin in ASA-PS I-II patients lower than 107 g/L were found. Besides, there was no critical result (based on our laboratory protocols and thresholds, data not shown) in any pathological test unexpected or not. There was not any change in anaesthetic management for any patient due to these findings.

In 2016 we performed a total amount of routine POTs on 5975 patients (23,900 total tests). This represented an annual total expense of 131,450 €.

## Discussion

The degree of inappropriateness in ordering laboratory routine POTs in our hospital is quite high due not only to the very low rate of pathological findings but to the very low percentage of unexpected pathological tests based on previous results within 6 months (2.1%). Even in more severe patients (ASA-PS III-IV), the rate of unexpected pathological results is unsubstantial (0.8%). This could be explained by the fact that these patients, due to their severity, are far more monitored from a biochemical point of view than less severe patients having more laboratory results on their medical records in the last 6 months. Besides, the lack of any change in anaesthetic management due to these findings makes that performing battery of tests on a routine basis without clinical indication an inefficient and low value activity.

Previous studies have demonstrated that the frequency of pathological laboratory test results involving outpatients is very low and 60 - 75% of patients would have not required any test if medical records were checked and clinical evaluations were performed (*1*, *2*, *5*, *6*). A very recent updated NICE (UK National Institute for Health and Clinical Excellence) guidance regarding routine POTs for elective surgery not only suggests including the results of tests undertaken in primary care when referring people for surgical consultation in order to avoid unnecessary repetition but emphasizes the lack of evidence that routine tests either improve or worsen postoperative outcome. More specifically, a systematic review of the literature has recommended avoiding routine coagulation screening before procedures to predict bleeding risk because unexpected coagulation defects are uncommon (*3*). In those cases, it is more preferable to review medical records looking for bleeding abnormalities.

The Practice Advisory for Preanesthesia Evaluation strongly recommends not to order POTs routinely (*1*). In fact, they may be ordered, required or performed on a selective basis for purposes of guiding or optimizing preoperative management. Instead of a shotgun approach, where “preop status” is just considered a specific clinical purpose, the indications for such laboratory testing should be based first on information from medical records, patient interview, physical examination and type and invasiveness of the surgical procedure (*7*).

In terms of economical cost and assuming that approximately 74% of our annual POTs were performed inappropriately in ASA-PS I-II patients, we could have saved in 2016 up to 97,273 €. An additional restriction in ASA-PS III-IV with previous ORI results within 6 months could have increased these savings. In fact, our next step will involve the introduction of a clinical decision supporting rule (CDSR) through electronical request when ordering POTs in order to report and/or cancel tests when previous pathological results within 6 months are expected or known. Our study nowadays stands as one of the studies with more patients included as far as we know (*8*, *9*).

In the light of the data obtained, it seems obvious that changes in pre-anaesthesia evaluation schemes must be introduced, moving towards a more “selective” approach regarding POTs. In summary, tests have to be always ordered (by surgeons and/or anaesthetists) after a previous consideration of specific information obtained from several medical sources and only if the information obtained will result in changes in the preoperative management of the patient (*1*, *7*). The main goal is to provide cost-effective quality patient care. Interventions to educate and improve collaboration between specialists, CDSRs through electronical request and implementation of consensus guidelines can avoid waste of human resources and time, maximizing efficiency (*10*).
